# The effect of periodontal therapy on C-reactive protein, endothelial function, lipids and proinflammatory biomarkers in patients with stable coronary artery disease: study protocol for a randomized controlled trial

**DOI:** 10.1186/1745-6215-14-283

**Published:** 2013-09-06

**Authors:** Marco Aurélio Lumertz Saffi, Mariana Vargas Furtado, Márlon Munhoz Montenegro, Ingrid Webb Josephson Ribeiro, Cassio Kampits, Eneida Rejane Rabelo-Silva, Carisi Anne Polanczyk, Cassiano Kuchenbecker Rösing, Alex Nogueira Haas

**Affiliations:** 1 Hospital de Clínicas de Porto Alegre, Cardiology Division, School of Medicine, Federal University of Rio Grande do Sul, Ramiro Barcelos 2350, Porto Alegre, Brazil; 2 Institute for Health Technology Assessment (IATS-CNPq), Ramiro Barcelos 2350, Porto Alegre, Brazil; 3 Faculty of Dentistry, Periodontology, Federal University of Rio Grande do Sul, Ramiro Barcelos 2492, Porto Alegre 90035-003, Brazil

**Keywords:** Periodontal diseases, Cardiovascular diseases, C-reactive protein, Non-surgical periodontal therapy, Coronary artery disease, Randomized controlled trial, Endothelial function, Lipids

## Abstract

**Background:**

Scarce information exists regarding the preventive effect of periodontal treatment in the recurrence of cardiovascular events. Prevention may be achieved by targeting risk factors for recurrent coronary artery disease (CAD) in patients with previous history of cardiovascular events. The aim of this trial is to compare the effect of two periodontal treatment approaches on levels of C-reactive protein, lipids, flow-mediated dilation and serum concentrations of proinflammatory and endothelial markers in stable CAD patients with periodontitis over a period of 12 months.

**Methods/design:**

This is a randomized, parallel design, examiner blinded, controlled clinical trial. Individuals from both genders, 35 years of age and older, with concomitant diagnosis of CAD and periodontitis will be included. CAD will be defined as the occurrence of at least one of the following events 6 months prior to entering the trial: documented history of myocardial infarction; surgical or percutaneous myocardial revascularization and lesion >50% in at least one coronary artery assessed by angiography; presence of angina and positive noninvasive testing of ischemia. Diagnosis of periodontitis will be defined using the CDC-AAP case definition (≥2 interproximal sites with clinical attachment loss ≥6 mm and ≥1 interproximal site with probing depth ≥5 mm). Individuals will have to present at least ten teeth present to be included. One hundred individuals will be allocated to test (intensive periodontal treatment comprised by scaling and root planing) or control (community periodontal treatment consisting of one session of supragingival plaque removal only) treatment groups. Full-mouth six sites per tooth periodontal examinations and subgingival biofilm samples will be conducted at baseline, 3, 6 and 12 months after treatment. The primary outcome of this study will be C-reactive protein changes over time. Secondary outcomes include levels of total cholesterol, LDL-C, HDL-C, triglycerides, IL-1β, IL-6, TNFα, fibrinogen, ICAM-1, VCAM-1 and E-selectin. These outcomes will be assessed at all time points over 12 months. Flow-mediated dilation will be assessed at baseline, 1, 3 and 6 months after periodontal therapy.

**Discussion:**

This trial will provide new evidence regarding the effect of periodontal treatment on risk markers for recurrence of cardiovascular events in stable coronary artery disease patients.

**Trial registration number:**

ClinicalTrials.gov Identifier, NCT01609725

## Background

Cardiovascular diseases (CVD) are still considered the main cause of mortality and morbidity all over the world [1]. In the last years, efforts have been made to define the inflammatory pathways that lead to cardiovascular events aiming to define more effective therapeutic and preventive strategies. In this regard, it has been demonstrated that many proinflammatory biomarkers play an important role in the cascade of events observed in CVD, mainly C-reactive protein (CRP) and other molecules such interleukins, fibrinogen and adhesion molecules [2-4].

Periodontal diseases have been considered a probable risk factor for CVD with a great amount of evidence from observational studies associating the two conditions [5-10]. The increase in the risk of coronary artery disease in periodontal patients is estimated to be 24% higher after adjusting for important confounding factors [11]. Despite the considerable amount of evidence associating the two conditions, there is still lack of interventional studies to better elucidate the effect of periodontal treatment on the prevention of CVD [10,12].

The biological plausibility linking periodontal diseases to CVD is supported by the findings that periodontal inflammation and infection lead to an increase in blood levels of the above-mentioned important biomarkers related to CVD. For instance, levels of CRP are higher in periodontitis compared to healthy patients, and reduction of this marker is observed after periodontal therapy in otherwise healthy individuals [13,14]. Interleukin 1β, interleukin 6, fibrinogen and other proinflammatory biomarkers have also been found in elevated levels in healthy patients with periodontitis [15,16]. It has also been demonstrated that periodontal disease may negatively influence endothelial function directly or indirectly [17-20].

Although there are some interventional studies evaluating the systemic effects of periodontal therapy [14], there is little information regarding the impact of periodontal treatment on the recurrence of cardiovascular events. Most of the studies have been conducted with otherwise healthy patients with periodontitis, limiting the applicability of the findings to patients suffering from CVD. To the best of our knowledge, there are no randomized controlled trials published to date assessing the preventive effect of periodontal therapy in true endpoints of cardiovascular disease, i.e., major cardiovascular events such as myocardial infarction and stroke. By reviewing the literature, we were able to find two small interventional studies showing that periodontal treatment might reduce some CVD blood risk markers. One of the studies demonstrated that CRP and hemostatic factors improved after periodontal treatment in a small group of 18 non-smokers with a history of a recent CVD event [21]. Another study evaluated hypertensive patients with periodontitis and showed that periodontal therapy might reduce levels of CRP and fibrinogen [22].

Recently, a study protocol of a short-term randomized controlled trial about the effect of periodontal therapy on endothelial function and some blood biomarkers for CVD was published [23]. The study is planned to be conducted with systemically healthy individuals with periodontitis and will provide data for primary prevention of CVD.

The aim of this article is to describe the protocol of a randomized controlled trial that was designed to compare the effect of intensive and community periodontal treatments on levels of C-reactive protein, lipids, flow-mediated dilation and serum concentrations of proinflammatory and endothelial markers in stable coronary artery disease patients with periodontitis over a 12-month period.

## Methods/design

### Study design and centers

This is a randomized, parallel design, examiner-blinded, controlled clinical trial. This study will be conducted at the School of Dentistry of the Federal University of Rio Grande do Sul and at the University Hospital of Porto Alegre in Brazil. Cardiovascular patients will be recruited at the Ischemic Heart Disease Clinic at the university hospital. Blood samples and laboratory analyses will be conducted at the Clinical Research Center at the university hospital. Flow-mediated dilation will be performed at the sector of non-invasive methods of the university hospital. Periodontal clinical examinations and treatments will be conducted at the Periodontal Department of the School of Dentistry.

### Inclusion and exclusion criteria

Individuals from both genders, 35 years of age and older, with concomitant diagnosis of CAD and periodontitis will be selected for the study. The diagnosis of CAD will be recalled from the history of acute coronary syndrome episodes or percutaneous/surgical revascularization [24] recorded at the hospital files. Specifically, CAD will be defined as the occurrence of at least one of the following events 6 months prior to entering the trial: documented history of myocardial infarction; surgical or percutaneous myocardial revascularization and lesion >50% in at least one coronary artery assessed by angiography; presence of angina and positive noninvasive testing of ischemia.

Diagnosis of periodontitis will be defined using the CDC-AAP case definition [25]. Individuals will be classified as having severe chronic periodontitis in the presence of ≥2 interproximal sites with clinical attachment loss ≥6 mm and ≥1 interproximal site with probing depth ≥5 mm in non-adjacent teeth. Moreover, included individuals have to present at least 12 teeth.

Individuals will be excluded from the study if they use antibiotics or anti-inflammatory drugs during the follow-up period of the study.

### Ethical considerations

All participants will read and sign an informed consent before entering the study. The study protocol was approved by the Institutional Review Boards of the university hospital (protocol no. 12–265) and the Federal University of Rio Grande do Sul (protocol no. 18341). The study will be conducted according to the principles of the Declaration of Helsinki for human studies and to the Brazilian legislation for human studies of the Ministry of Health.

### Cardiovascular care

This study will be conducted with stable coronary artery disease patients who will be receiving cardiovascular care for at least 6 months at the Ischemic Heart Disease Clinic, a tertiary care cardiovascular clinic, at the University Hospital of Porto Alegre. Cardiovascular care provided in this tertiary clinic includes medication and counseling. The staff of the clinic comprises cardiologists, nurses, nutritionists and physiotherapists. The protocol of cardiovascular care in this clinic includes statins for the majority of the patients. When appropriate, oral hypoglycemic, insulin, acetylsalicylic acid and antihypertensive drugs are also prescribed. Counseling includes mainly health-related lifestyle behavioral modifications such as daily exercise, smoking cessation and dietary therapy. The mean follow-up time of the patients that attend the clinic is 5 years.

### Sample size

The sample size of the present trial was estimated using a statistical software (G*Power 3 for Macintosh). The change in serum levels of C-reactive protein was considered the main outcome for sample size estimation. It was estimated that 42 individuals in each treatment arm will be necessary to find reductions in C-reactive protein of 1.5 mg/l (standard deviation = 1.5) and 0.5 mg/l (standard deviation = 0.5) in the test and control groups, respectively. Alpha and beta errors of 5% and 20%, respectively, were used in the estimation. An attrition rate of 20% is expected during the study; consequently, 50 patients in each group will be included.

### Interventions

The test group (TG) will consist of intensive periodontal treatment comprising one session of supragingival scaling and personalized oral hygiene instructions, followed by one to four sessions of subgingival scaling and root planing (SRP) by quadrant, under local anesthesia, in a period of 14 days. Individuals will be followed monthly during the first 6 months and at each 3 months until the end of the 12-month study period. In the follow-up sessions, professional plaque removal, oral hygiene instructions and reinforcement will be provided.

The control group (CG) will receive community periodontal treatment similar to that provided by the Brazilian public health system, consisting of only one session of supragingival scaling followed by standard oral hygiene instructions.

Treatments will be performed by two experienced periodontists (I.W.J.R. and C.K.). Before the beginning of the study, the two clinicians will undergo a period of training and discussion of the therapeutic approaches with the aim of standardization of the test and control interventions.

### Study protocol

**Figure 1:**
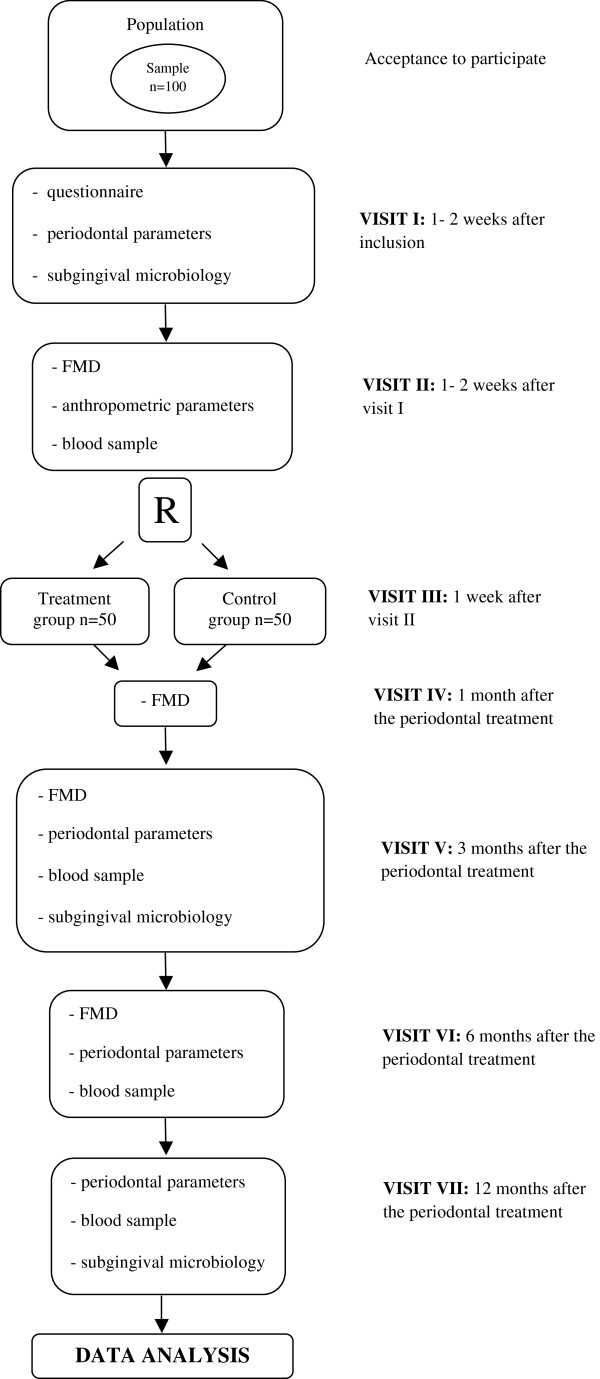
Flowchart of the study participation and examinations.

Eligible individuals will be invited to follow the protocol of visits described below (Figure 1):

•Visit I: application of a structured questionnaire, general clinical evaluation, baseline periodontal examination and subgingival microbiological samples.

•Visit II: baseline flow-mediated dilation, anthropometric assessment and blood samples.

•Visit III: Start of periodontal treatment according to randomization. After this visit, periodontal therapy will be conducted according to randomization.

•Visit IV (30 days after the last session of periodontal treatment): first follow-up flow-mediated dilation assessment.

•Visit V (3 months after the last session of periodontal treatment): second follow-up flow-mediated dilation assessment, first follow-up blood samples, periodontal clinical examination and subgingival microbiological samples.

•Visit VI (6 months after the last session of periodontal treatment): third follow-up flow-mediated dilation assessment, second follow-up blood samples and periodontal clinical examination and subgingival microbiological samples.

•Visit VII (12 months after the last session of periodontal treatment): final follow-up blood samples, periodontal clinical examination and subgingival microbiological samples.

### Randomization

Stratified randomization will be conducted. Individuals will be stratified into localized or generalized periodontitis groups using a cutoff point of 30% of teeth with clinical attachment loss ≥ 6 mm since the extent of disease may be a prognostic factor for the response to non-surgical periodontal treatment.

A specific program (randomization.com) for allocation in test and control groups will be used by generating a random numbers sequence. Randomization will be conducted in blocks of 20 individuals after attributing codes to each participant. An external assistant not involved in the study will conduct randomization to warrant allocation concealment. Codes will be kept until the end of analyses to maintain blindness of the examiner and statistician.

### Blood markers

Each individual will provide two 10-ml samples of blood collected by a trained nurse from the antecubital fossa. Fasting blood samples will be obtained between 7:00 a.m. and 12:00 p.m. to control for possible diurnal variations. Blood samples for traditional cardiovascular blood markers will be immediately centrifuged and analyzed. Blood samples used for proinflammatory and endothelial markers will be centrifuged at 4°C and 4.000 rpm for 10 min (ALC PK 120 R, ALC International, Milan, Italy). The serum will be sampled and stored in Eppendorf tubes at −80°C until analysis.

Traditional cardiovascular blood risk markers to be assessed will be glucose, glycated hemoglobin, triglycerides (TG), total cholesterol, and high- and low-density lipoprotein cholesterol (HDL-C and LDL-C). The following proinflammatory markers will be assessed: C-reactive protein, interleukin 1β (IL-1β), IL-6, tumor necrosis factor α (TNFα) and fibrinogen. ICAM-1, VCAM-1 and E-selectin will be the measured to assess the endothelial function. These markers will be measured at baseline, 3, 6 and 12 months after treatment.

High-sensitive CRP, glucose, TG, total cholesterol and HDL-C will be measured by automated enzymatic colorimetric methods (ADVIA 1800, Siemens, Germany) following the manufacturer’s instructions. CRP will be measured using the intensified immunoturbidimetry by latex (CRP_2). Glucose will be obtained using the glucose-hexokinase method II (GLUH). Total cholesterol will be dosed by the colorimetric enzymatic method (CHOL_2) with cholesterol-esterase and cholesterol oxidase followed by a Trinder type endpoint. Triglycerides will be measured by the Trinder GPO method. HDL-C will be assessed by the HDL-*Directo* (HDL-D) using the principles of elimination/catalase. LDL-C will be calculated using the Friedwald formula [LDL-C = total cholesterol – (HDL-C + TG/5)]. Non-HDL-C will be calculated by the subtraction of HDL-C from total cholesterol. Very low density lipoprotein cholesterol (VLDL-C) will be calculated dividing TG by 5. Glycated hemoglobin will be obtained by high-precision chromatography (Merck-Hitachi L-9100, Merck, Germany).

Proinflammatory and endothelial function blood markers will be measured using specific kits for Luminex (Milliplex map human panels, EMD Millipore Corp., Billerica, MA, USA).

### Periodontal clinical examination

The periodontal clinical examinations will be conducted by one single calibrated examiner to assess the periodontal status of each participant. Clinical examinations will be performed using a 10-mm round-tip manual Williams periodontal probe. All permanent teeth, excluding the third molars, will be examined at six sites per tooth (disto-buccal, mid-buccal, mesio-buccal, disto-palatal, mid-palatal, mesio-palatal). Visible plaque (VP), gingival recession (GR), periodontal probing depth (PD) and bleeding on probing (BOP) will be recorded. Clinical attachment loss will be obtained by the sum of GR and PD.

### Subgingival biofilm

The supragingival biofilm will be removed using sterile cotton and curettes. Relative isolation will be conducted in the area to be sampled. The subgingival biofilm samples will be obtained using no. 30 sterile paper points inserted for 1 min in the pocket. Paper points will be stored in one Eppendorf tube at −20°C until analyzed. The four deepest sites of each individual will be sampled.

The presence of periodontopathogenic bacteria in the subgingival biofilm will be assessed by real-time polymerase chain reaction (RT-PCR). Three species will be evaluated (*T. forsythia*, *P. gingivalis* and *T. denticola*).

### Endothelial function

Non-invasive measurements of endothelial function will be conducted by flow-mediated dilation (FMD) of the brachial artery using bidimensional ultrasound equipment. Individuals will have to be rested and will lay in a controlled-temperature room. All vasodilation medication will have to be interrupted for at least 4 h before the examination, if possible. Individuals will also be advised to refrain from exercising, drinking caffeine and smoking for at least 4 h before the examination. The examination will start after 15 min of rest in the supine position with the arms in a comfortable position. An image of the brachial artery will be obtained above the antecubital fossa in a longitudinal plane. The flow will be monitored with the Doppler positioned at a 65^o^ angle, and images will be obtained between the lumen and the wall of the vein. A sphygmomanometer will be inflated at the right forearm for 5 min, at least 50 mmHg above the systolic pressure, for measurement of reactive hyperemia. Images will be established 30 s before and until 2 min after sphygmomanometer deflation, synchronized with the “R” wave of the electrocardiogram. FMD will be expresses as the relative variation of the brachial diameter in the hyperemic phase and defined as [(post-hyperemic diameter – baseline diameter)/baseline diameter] × 100.

After 15 min of the reactive hyperemic measurement and artery diameter normalization, the baseline artery diameter will be measured again. In the sequence, a dose (spray) of sublingual NTG will be administered to evaluate dependent endothelial vasodilation with images acquired after 5 min [26].

### Demographics, behavioral and clinical variables

A structured questionnaire will be applied to all participants to obtain demographic and behavioral data. Age, socioeconomic level, education, medication use, smoking habits and frequency of physical activities will be recorded. Individuals will be examined clinically to measure height, weight and blood pressure. Body mass index (BMI) will be calculated dividing the weight by the square of the height.

### Primary outcome

The primary outcome of the present trial will be changes in serum concentrations of C-reactive protein.

### Secondary outcomes

Secondary outcomes will include:

•Non-invasive measurements of endothelial function (FMD);

•Traditional cardiovascular risk markers (total cholesterol, LDL-C, HDL-C and triglycerides);

•Proinflammatory biomarkers (IL-1β, IL-6, TNFα and fibrinogen);

•Endothelial function biomarkers (ICAM-1, VCAM-1 and E-selectin).

### Independent/exposure variables and confounding

The main exposure variable will be composed by test and control periodontal treatments. The impact of each treatment on the primary and secondary outcomes will be evaluated controlling for the following confounding factors: age, gender, BMI, physical activity, glycated hemoglobin, use of oral hypoglycemic drugs, smoking exposure and subgingival microbiota.

### Statistical analyses

Continuous variables will be described using means and standard deviations or median and range in case of asymmetric distribution of data. Categorical variables will be presented using frequency distribution. An intention-to-treat approach will be used in the analysis of this trial taking into consideration all dropouts during the follow-up period.

Univariate analyses will be conducted using chi-square and *t* tests for independent samples. Multivariate models will be fitted using generalized estimating equations to compare changes in each outcome according to test and control periodontal treatment groups controlling for confounding factors. *P* values <0.05 will be considered statistically significant. A statistical package (Stata 12 for Macintosh, STATA Corp., College Station, TX, USA) will be used. The individual will be considered the unit of analysis.

### Trial status

This is an ongoing trial. One first block of randomized patients is receiving the interventions, and more participants are being recruited.

## Competing interests

The authors declare they do not have any competing interest related to this study.

## Authors’ contributions

MALS will conduct the flow-mediated dilation measurements and drafted this manuscript. MVF conceived the study and drafted this manuscript. MMM will conduct all the periodontal examinations and drafted this manuscript. IWJR will conduct the microbiological analyses and drafted this manuscript. CK will conduct periodontal treatments and drafted this manuscript. ERRS conceived the study and drafted this manuscript. CAP is Head of the Cardiovascular Clinic, conceived the study and drafted this manuscript. CKR is Head of Periodontology, conceived the study and drafted this manuscript. ANH will conduct all statistical analyses, conceived the study and drafted this manuscript. All authors read and approved the final manuscript.

## Funding

The present trial is receiving support from two grants, one from the Brazilian Ministry of Science and Technology (CNPq 476387/2010-8) and another from the Research Support Agency from Rio Grande do Sul State (FAPERGS 1008214). Funding is also being provided by the Funding for Research and Events from the University Hospital of Porto Alegre.
